# Remote Ischemic Conditioning in Ischemic Stroke and Myocardial Infarction: Similarities and Differences

**DOI:** 10.3389/fneur.2021.716316

**Published:** 2021-10-26

**Authors:** Luigi F. Saccaro, Alberto Aimo, Michele Emdin, Fernando Pico

**Affiliations:** ^1^Neurology and Stroke Care Unit, Versailles Hospital, Le Chesnay, France; ^2^Institute of Life Sciences, Scuola Superiore Sant'Anna, Pisa, Italy; ^3^Cardiology Division, Fondazione Toscana Gabriele Monasterio, Pisa, Italy; ^4^Neurology Department, Versailles Saint-Quentin-en–Yvelines and Paris Saclay University, Versailles, France

**Keywords:** ischemic stroke, remote ischemic conditioning (RIC), neuroprotection, cardiac protection, myocardial infarction

## Abstract

Acute myocardial infarction and ischemic stroke are leading causes of morbidity and mortality worldwide. Although reperfusion therapies have greatly improved the outcomes of patients with these conditions, many patients die or are severely disabled despite complete reperfusion. It is therefore important to identify interventions that can prevent progression to ischemic necrosis and limit ischemia-reperfusion injury. A possible strategy is ischemic conditioning, which consists of inducing ischemia – either in the ischemic organ or in another body site [i.e., remote ischemic conditioning (RIC), e.g., by inflating a cuff around the patient's arm or leg]. The effects of ischemic conditioning have been studied, alone or in combination with revascularization techniques. Based on the timing (before, during, or after ischemia), RIC is classified as pre-, per-/peri-, or post-conditioning, respectively. In this review, we first highlight some pathophysiological and clinical similarities and differences between cardiac and cerebral ischemia. We report evidence that RIC reduces circulating biomarkers of myocardial necrosis, infarct size, and edema, although this effect appears not to translate into a better prognosis. We then review cutting-edge applications of RIC for the treatment of ischemic stroke. We also highlight that, although RIC is a safe procedure that can easily be implemented in hospital and pre-hospital settings, its efficacy in patients with ischemic stroke remains to be proven. We then discuss possible methodological issues of previous studies. We finish by highlighting some perspectives for future research, aimed at increasing the efficacy of ischemic conditioning for improving tissue protection and clinical outcomes, and stratifying myocardial infarction and brain ischemia patients to enhance treatment feasibility.

## Introduction

Myocardial infarction (MI) and ischemic stroke are leading causes of morbidity and mortality (1, 2). Both conditions have an acute onset and are due to blood vessel occlusion leading to a certain extent of ischemic necrosis.

MI usually follows thrombotic occlusion of a coronary artery due to a vulnerable plaque rupture. Ischemia-dependent mitochondrial and metabolic alterations lead to systolic function depression and, when persistent, to cardiomyocyte necrosis followed by tissue scarring (3). Similarly, ischemic stroke results from a lack of blood flow to the brain, which reduces oxygen, glucose and nutrient supply, as well as, secondarily, catabolite removal. Blood deprivation is typically caused by large artery atherosclerosis, cardiac embolic events, small vessel occlusion, or stroke of other etiologies (4).

In cardiac, as in brain ischemia, there is a clear major effect of early restoration of blood flow through reperfusion therapies on outcomes. These include pharmacologic (i.e., fibrinolytic therapy) or mechanical interventions, namely primary percutaneous coronary intervention (PPCI) or endovascular thrombectomy. More than 90% of MI patients receive reperfusion therapy against ~10% of acute ischemic stroke patients (5). Among the factors that account for this difference, the different time windows from symptom onset for beneficial reperfusion treatment should be taken in account. These are usually <12 h (or between 12 and 48 h in some patients with persisting symptoms) for fibrinolytic therapy for ST-segment elevation MI (STEMI), 4.5 h (9 h in some patients with radiological signs of salvageable brain tissue) for thrombolysis of brain ischemia, and <24 h for mechanical thrombectomy in brain ischemia. Furthermore, an arterial occlusive thrombus accessible to catheter-based intervention is found in about 90% of MI patient, but only about half of computed tomography (CT) angiograms performed for acute ischemic stroke (5). Indeed, while there are few contraindications to coronary catheter-based interventions, reperfusion therapies for ischemic stroke are absolutely contraindicated if there is intracranial bleeding or advanced ischemia. Another reason possibly accounting for the difference in the percentage of patients that receive reperfusion therapy between ischemic stroke and MI may be that time to treatment is often longer in the former condition (6). Finally, biomarkers of brain ischemia are missing (while troponins are widely used in cardiovascular medicine), and neurological diagnostic methods [CT or magnetic resonance imaging (MRI)] are expensive, time-consuming, and not routinely performed outside hospital or at the bedside (contrary to an electrocardiogram) (5), although technological advances (such as mobile CT or bedside MRI) may change this (7, 8).

Patients with MI or ischemic stroke who receive successful reperfusion therapies are still exposed to certain risks, as reperfusion itself is an important determinant of end-organ damage. Indeed, ischemia triggers a vicious cycle of cell death, inflammation, and oxidative stress, which is perpetuated by reperfusion and may increase the extent of infarction in otherwise viable brain or cardiac tissue (9, 10), also in association with cerebral edema and blood–brain barrier disruption (11). Reperfusion injury is much more common and more often leads to hemorrhagic transformation in brain infarct than MI (5). Intracranial hemorrhage exposes the patient to life-threatening intracranial hypertension, with the risk of brain herniation (5).

The risk of these detrimental effects is usually counterbalanced by the fact that reperfusion therapies can save the border (or marginal) zone of MI or the ischemic penumbra in ischemic stroke, if administered promptly. The border zone (or penumbra in ischemic stroke) is the salvageable tissue around the ischemic core, in which reduced blood flow causes loss of cell function with normal structural morphology, before irreversible damage, which occurs instead in the ischemic core (12, 13). However, the recanalization rate with thrombolysis in brain ischemia is lower than with endovascular thrombectomy (14). Penumbral salvage becomes more likely with endovascular thrombectomy, which considerably improves clinical outcomes. Despite this, only about half of successful thrombectomies lead to patients' functional independence (15), mainly because the ischemic core is already too large at the time of recanalization. This may partially explain why most stroke patients are still disabled 3 months after treatment (15). As for MI patients, despite the fact that timely PPCI is associated with better outcomes than fibrinolysis, a significant number of patients with reperfused STEMI display the no-reflow phenomenon, which predicts a worse outcome, specifically a greater risk of ventricular wall rupture and arrhythmias, adverse ventricular remodeling with heart failure development, and cardiac death (3).

Reducing the burden of cardiac and cerebral ischemia-related death disability requires the identification of interventions able to “freeze the penumbra,” i.e., prevent the growth of the necrotic ischemic core until partial or complete reperfusion, as well as techniques to protect the ischemic tissue from subsequent reperfusion injury (12, 13, 16). However, interventions that aim to improve ischemic stroke and MI prognosis have, so far, shown an inconsistent benefit (17–20). Alternative cytoprotective strategies are being studied (21), but strong evidence on the efficacy of any proposed mechanism is lacking.

An interesting paradigm may be ischemic conditioning (22), first described in 1986 by Murry et al. (23) in the setting of experimental MI. In ischemic conditioning, transient, intermittent ischemia without necrosis is induced either in the organ undergoing spontaneous ischemia (i.e., conventional conditioning), or at a distance from the affected organ [i.e., indirect or remote ischemic conditioning (RIC)]. According to the timing of the intervention (before, during, or after ischemia), remote ischemic pre-, per-/peri-, and post-conditioning, respectively, can be defined. Pre-conditioning has been defined as “an adaptive process of endogenous protection in which small, sublethal doses of a harmful agent protect the organism against a later lethal dose of the same agent” (24). In the settings of acute MI and ischemic stroke, per- or post-ischemic conditioning is more easily realized. This can be achieved by the application of an inflatable cuff around the patient's arm or leg (22). After extensive evaluation in the field of cardiac ischemia, the paradigm of RIC has recently been translated to ischemic stroke (20), and seems to apply to other organs and tissues. Plasma dialysate obtained from animals and humans treated with RIC has been shown to reduce MI size after ligation of a coronary artery and subsequent reperfusion in isolated heart preparations (25, 26), indicating that the effect may be mediated (at least partially) by humoral substances released from the tissues exposed to intermittent ischemia. In animal models, remote ischemic pre- and post-conditioning have been shown to reduce MI size and biomarkers of myocardial necrosis (27–29). Similarly, in rat models of middle cerebral artery occlusion, brain infarct size was reduced by remote pre-conditioning (30).

This review aims to discuss the existing clinical evidence on RIC in brain and myocardial ischemia. We will first of all synthetically recapitulate potential mechanisms of RIC, then discuss main clinical findings in MI, first, and in ischemic stroke, then, highlight the differences and similarities, as well as future perspectives and therapeutic implications.

## Putative Mechanisms mediating pre-, per-, and post-RIC Effects in Pre-clinical Models

**Figure 1:**
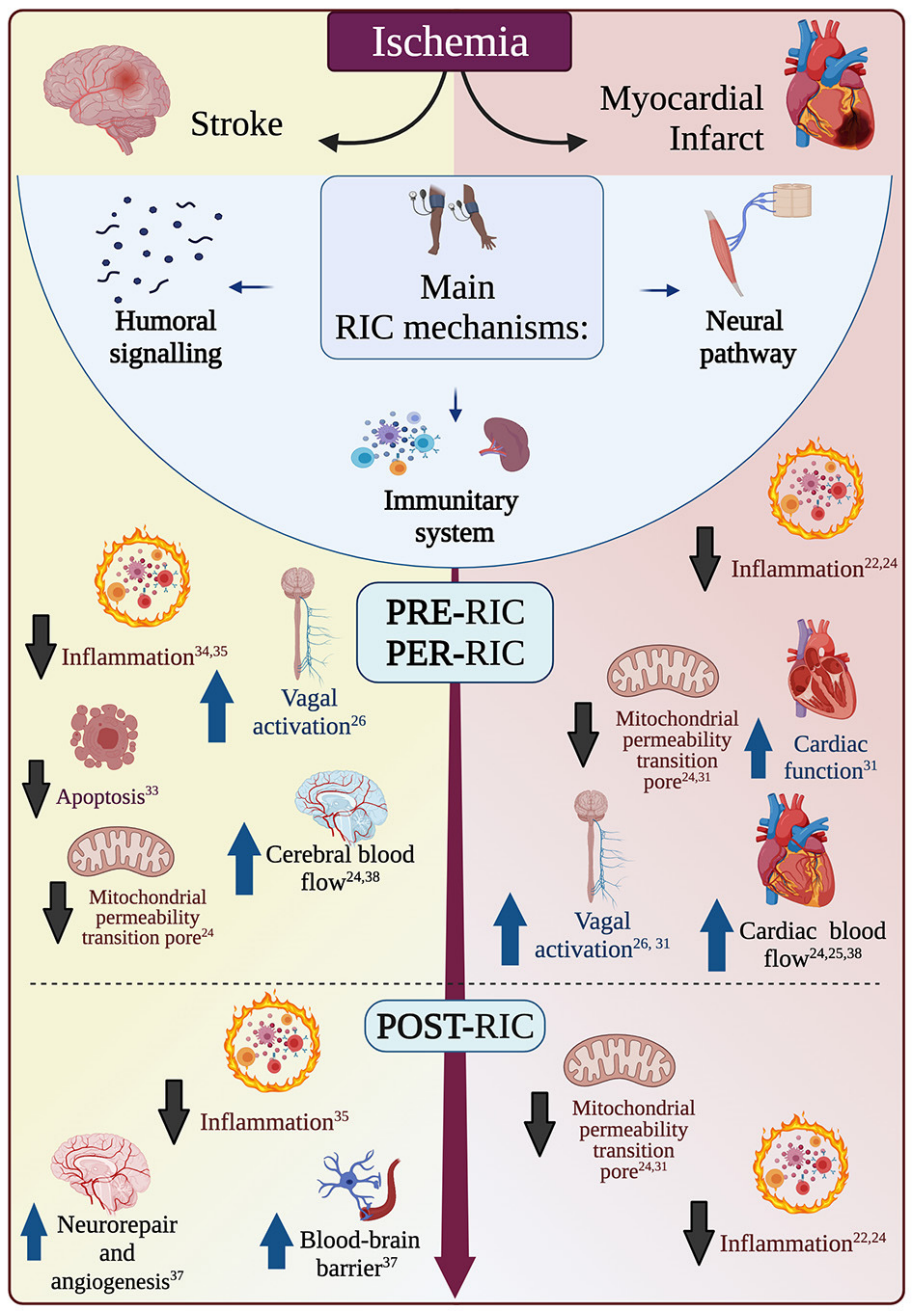
Main mechanisms of remote ischemic conditioning (RIC). RIC can be performed before (pre-RIC), during (per-RIC), or after (post-RIC) an ischemic event (ischemic stroke or myocardial infarction). RIC effects are mostly mediated by humoral signaling, neural pathways, or modulation of systemic immune system. These different pathways generate many effects that have different importance in the setting of myocardial infarction (red, on the right) or ischemic stroke (yellow, on the left), while some effects are likely of similar importance in both conditions (in the white area in the middle). While the same mechanisms may have different importance before, during or after an ischemic event, there is not enough literature to attribute each mechanism to a certain phase only. Upward blue arrows indicated increase and enhancement, while downward black arrows indicate reduction and inhibition.

The exact mechanisms of this remote organ protection from ischemia are unknown and could differ between pre-, per-, and post-conditioning. While it is beyond the scope of this review to detail all putative mechanisms, we will briefly recapitulate the main ones in the paragraphs that follow and in Figure 1. For further details on potential cardioprotective mechanisms, please see Aimo et al. (31), while, for further details on putative neuroprotective mechanisms, please see reviews by Basalay et al. and Chen et al. (26, 32). Of note, more mechanisms may be involved at the same time, and they may be more or less important depending on the setting of pre-, per or post-conditioning. Further investigations of the protective mechanisms of RIC are needed to aid a safe and effective translation to clinical practice. Muscle ischemia may release autocoids (e.g. bradykinin, opioids or adenosine), which might enter the systemic circulation (humoral hypothesis), or locally activate somatic nerve afferents (neural hypothesis). Supporting the neural hypothesis, in several preclinical models of brain infarction peripheral nerve block (either pharmacological or by resection) reduced or abolished RIC neuroprotective effects (30). Further, RIC-dependent vagal nerve activation may have an anti-inflammatory effect mediated by the spleen and liver via the cholinergic anti-inflammatory signaling (24). However, while it is logical to think that protective biomarkers that may be generated by RIC are directly available for the cardiac tissue, it is far from clear whether such molecules can cross the blood-brain barrier and reach the cerebral tissue. Most of the pathways activated by RIC are believed to ultimately affect the mitochondria, preventing for example the formation of the mitochondrial permeability transition pore (MPTP) or leading to the cytoprotective nitrosylation of key mitochondrial proteins, such as those forming complex I and complex IV. This could reduce the generation of mitochondrial reactive oxygen species. Finally, it ought to be noted that RIC has been shown to improve cardiac function, which, in turn, is positively correlated with cerebral blood flux, and could therefore represent yet another mediator of RIC-neuroprotective effects (33).

### Putative Mechanisms Mediating Pre- and Per-RIC Effects in Myocardial Infarction

The effects of pre-RIC-induced cardioprotection may be mediated by humoral factors acting through the systemic circulation (e.g., stromal cell-derived factor-1, interleukin-10, microRNA 144, and nitrite, which, for example, may favor vasodilation); by nervous reflexes or neurogenic transmission, through the autonomic fibers activated by, for example, adenosine or bradykinin (26, 31), or, as mentioned, by the effects on circulating immune cells (e.g., inhibition of leucocyte CD11b expression and a reduced number of cardiac macrophages and neutrophils), reducing inflammation, apoptosis, and oxidative stress (22, 24).

Per-RIC mechanisms are probably similar to pre-RIC ones, comprising humoral and neural autonomic pathways and vagal nerve activation (enhancing the sympathovagal balance), as well as inflammatory modulation (22, 24, 26, 31).

### Putative Mechanisms Mediating Post-RIC Effects in Myocardial Infarction

Contrary to pre- and per-RIC mechanisms, post-RIC effects do not seem to be mediated by vagal activation and the aforementioned humoral factors are likely to play a predominant role in reducing inflammation and apoptosis (26, 31).

### Putative Mechanisms Mediating Pre- and Per-RIC Effects in Ischemic Stroke

Pre-RIC may have neuro- and cardio- protective effects, reducing the damage of ensuing ischemia. These effects are believed to be mediated mainly by increase of cerebral blood probably mediated by induction of nitric oxide synthase (24) and reduction of inflammation (e.g., inhibition of leucocyte CD11b expression and a reduced number of cardiac macrophages and neutrophils) (22, 24), which is detrimental for ischemic tissue. This anti-inflammatory action may also be mediated by vagal nerve activation triggered by RIC.

In any case, at local levels, pre-RIC-induced neuroprotection has been found to depend on inhibition of inflammatory response and apoptosis in animal models of brain ischemia (34, 35).

Per-RIC effects are likely mediated by an increase of cerebral blood flow (24). Nitric oxide in particular may play an important role in enhancing cerebral blood flow, as it has been shown in animal models of ischemic pre- and per-conditioning (36). As pre-RIC, also per-RIC has been shown in animal models of brain ischemia to reduce ischemia-reperfusion-injury decreasing infarct size, brain edema and neurological deficit scores, through inhibition of pro-inflammatory signals, in particular the TLR4/NF-κ pathway (37).

### Putative Mechanisms Mediating Post-RIC Effects in Ischemic Stroke

Post-RIC effects may be, at least in part, mediated by endogenous neuroprotective and neurorepairing responses, such as increased local production of neuronal nitric oxide synthase (38), BDNF or endogenous opioids in the central nervous system, or stimulation of cerebral angiogenesis and inhibition of oxidative stress and inflammatory responses, possibly through vagal nerve activation (39). Speculatively, it might be hypothesized that vagal nerve activation modulates cerebral excitability, and this effect might play a role in improving recovery, but further research is needed on this point. Other potential mechanisms mediating post-RIC neuroprotection involve inhibition of apoptotic signals, alleviation of cerebral edema and enhancement of blood-brain barrier and neurovascular unit integrity (39).

## Remote Ischemic Conditioning in Myocardial Infarction

Building on the experimental results mentioned in the introduction, several studies have tested RIC in patients with STEMI. In these studies, RIC is delivered together with PPCI. Both peri- and post-ischemic conditioning, generally induced by four 5-min cycles of limb cuff inflation and deflation, were found to reduce the release of creatinine kinase-MB (CK-MB) (41) and high-sensitivity troponin T at different timepoints (42, 43), as well as infarct size (41, 44), microvascular obstruction (44), edema (41), and other markers of myocardial salvage (45). Also, peri-ischemic conditioning (four 5-min cycles of upper arm cuff inflation to 200 mmHg and deflation) associated with thrombolysis for STEMI reduced enzymatic markers of MI (46). In another study of 333 patients with suspected first acute MI undergoing PPCI, peri-ischemic conditioning with four cycles of 5-min inflations and deflations of a blood-pressure cuff had no effect on troponin T release nor infarct size (21). However, peri-ischemic conditioning improved myocardial salvage index, calculated as (area at risk–final infarct size)/area at risk (21). A randomized controlled trial of 151 STEMI patients did not find any additive effect of local ischemic post-conditioning (four cycles of 1-min inflations and 1-min deflations of the angioplasty balloon) to remote ischemic per-conditioning (three cycles of 5-min inflations to 200 mmHg and 5-min deflations of an upper-arm cuff) (47). The latter alone or the two combined had a similar effect in reducing peak CK-MB, the ratio of CK-MB area under the curve to myocardial area at risk, and the ratio of peak CK-MB to the area at risk (47). However, differences in CK-MB area under the curve between control, per-conditioning alone, and per-conditioning with post-conditioning were not statistically significant (47).

While these and other small randomized controlled trials have shown that RIC in addition to reperfusion therapies may blunt the release of myocardial necrosis enzymes and infarct size, or improve myocardial salvage in STEMI patients, results are heterogenous, and different biomarkers gave positive results in different studies. Furthermore, the aforementioned studies do not prove that RIC can improve clinical endpoints, such as mortality or heart failure. Other studies have tried to answer such questions, evaluating the effect of RIC on clinical outcomes in STEMI patients undergoing PPCI. A prospective randomized trial of 696 acute STEMI patients found that combined remote ischemic per-conditioning (three cycles of inflation of an upper-arm cuff for 5 min followed by deflation for 5 min) and post-conditioning (four cycles of 30-s balloon occlusions followed by 30 s of reperfusion) in addition to PPCI slightly reduced the rate of major adverse cardiac events (MACE) and heart failure development at a median of 3.6 years, although the study was not powered for detecting follow-up clinical outcomes (48). However, post-conditioning alone did not decrease MACE compared to controls who received PPCI alone (48). Similarly, two randomized controlled trials showed that remote ischemic per-conditioning in addition to PPCI improved long-term clinical outcomes in patients with STEMI (49, 50). However, these studies had a low statistic power and were not designed to prospectively detect differences in clinical outcomes between patients receiving RIC and controls (51). Furthermore, none of these trials showed an effect of RIC on MI size reduction (19, 49–51). On the contrary, a large, appropriately powered, international, prospective, randomized controlled trial of 5,401 patients with STEMI who underwent remote ischemic per-conditioning (three cycles of intermittent 5-min lower limb ischemia) did not find any effects on the incidence of MACE at 12 months (51). In this study Hausenloy et al. (51), used the RIC protocol that has been showed to be the most effective in experimental studies (52). Notably, it has been hypothesized that RIC might initiate a form of delayed protection, the clinical benefits of which may not manifest for 2 years or longer (19, 53, 54).

## Remote Ischemic Conditioning in Acute Ischemic Stroke

As in STEMI, pharmacological neuroprotective therapies for ischemic stroke have been disappointing (55). RIC has been shown to be effective in pre-clinical models of acute brain ischemia, both alone and in combination with revascularization therapies (56, 57). In humans, patients with transient ischemic attack (58–60) or peripheral ischemic vascular disease (61) before ischemic stroke have been proposed as possible “natural” models of ischemic pre-conditioning. In both of these populations, subsequent ischemic strokes were attenuated (smaller infarct volumes and lower disability and mortality), compared to ischemic stroke patients without clinical history of transient ischemic attack (58–60) or without peripheral ischemic vascular disease (61). However, these studies have some limitations, such as their retrospective design and the challenges of anamnestic identification of TIA, for instance.

In patients with symptomatic intracranial stenoses, RIC may reduce recurrent stroke, improve cerebral perfusion (62, 63), and decrease ischemic brain injury secondary to carotid artery stenting (64, 65). Nevertheless, evidence of the efficacy of RIC in acute ischemic stroke is lacking.

According to a recent systematic review (20), six studies that applied remote ischemic per-conditioning to acute ischemic stroke patients have been completed and 13 are ongoing. A marked heterogeneity exists in the number of participants, inclusion criteria, remote ischemic per-conditioning protocols, and main endpoints. In most cases, remote ischemic per-conditioning was applied to an unaffected upper limb, most often with an automated device, sometimes manually. Remote ischemic per-conditioning was only initiated in a pre-hospital setting in three trials (20): REMOTE-CAT, RESIST, and a study by Hougaard et al. (66).

The safety of RIC for brain ischemia patients undergoing thrombectomy or thrombolysis has been reported in different contexts by different groups (62, 64, 67, 68), including in octo- and non-agenarians (62), in patients with acute ischemic stroke (68), and in those undergoing thrombectomy (67). In particular, remote ischemic post-conditioning after thrombolysis has been investigated in a small, randomized trial in 30 patients (five 5-min cycles of inflation and deflation on the first day after thrombolysis, and twice each day for 6 consecutive days), which did not highlight any safety issues (64).

Trials evaluating RIC have mostly focused on surrogate markers of efficacy, such as neuroimaging findings (e.g., brain infarct size or tissue at risk for infarction based on cerebral perfusion). Alternatively, some studies have focused on circulating biomarkers. These include putative mediators of protective mechanisms of RIC [e.g., heat shock proteins, which have been associated with ischemic tolerance (69)]; markers of processes known to be detrimental in the course of brain ischemia, such as inflammatory proteins [e.g., C-reactive protein (CRP), serum amyloid protein (SAP), or tissue necrosis factor-α (TNF-α)]; or other possible markers of neuronal degeneration [e.g., S100B or matrix metalloproteinase-9 (70, 71)]. Thus, biomarkers have been used to assess the efficacy of a RIC protocol, either in reproducing the beneficial effects that RIC has shown in animal models (56), or in limiting inflammation and neuronal degeneration.

Most biomarkers did not change in patients with acute brain ischemia undergoing RIC in the RECAST trial (68). However, a significant increase in heat shock protein-27 and reductions in SAP and TNF-α levels were measured in patients undergoing RIC (four cycles of intermittent 5-min upper limb ischemia and reperfusion) compared to controls (68, 72). These results are particularly interesting as SAP levels before RIC displayed a moderate, yet significant correlation with worse clinical outcomes after brain ischemia, and were significantly reduced after RIC compared to before the intervention in intra-subject analyses (72). A decrease in high-sensitivity CRP in stroke patients undergoing ischemic conditioning has also been reported in a recent meta-analysis that included 13 clinical trials, for a total of 794 patients, mainly of Asiatic ethnicity (73).

Pre-hospital manual RIC (four cycles of intermittent 5-min upper limb ischemia and reperfusion) was found to reduce the radiological risk of brain tissue infarction in 443 patients (66). However, no difference in brain infarction volume growth at 24 h after symptom onset was identified in a multicenter study of 188 carotid ischemic patients who were randomized to lower-limb in-hospital remote ischemic per-conditioning (four cycles of 5-min ischemia and reperfusion) after initial MRI in addition to standard therapy or standard therapy alone (74). Other trials that are investigating the effect of RIC on radiological biomarkers, such as brain infarction volume [rtPA-RIC (NCT02886390); PROTECT I (NCT03915782); REVISE-2 (NCT03045055); RICE PAC (NCT03152799); REPOST (75)], are planned or ongoing.

Findings on clinical endpoints of RIC for acute ischemic stroke are even more limited. For example, in the aforementioned trial on 443 ischemic stroke patients by Hougaard et al. (66), neutral results were found: clinical neurological outcomes did not differ significantly between patients undergoing pre-hospital manual RIC and controls. Only four ongoing studies have clinical endpoints as primary outcomes: REMOTE-CAT, SERIC AIS, RESIST, and RICAMIS (20).

On the other hand, a metanalysis by Zhao et al. (73) found that remote ischemic post-conditioning may not only reduce the risk of recurrent stroke, but also the modified Rankin score (according to two studies) and the National Institutes of Health Stroke Scale score (despite significant heterogeneity in the trials that assessed this variable).

## Discussion

This review recapitulates the evidence that RIC reduces circulating biomarkers of myocardial necrosis, infarct size, and edema, although this effect does not appear to translate into better outcomes (19, 51) (Table 1). However, concerning ischemic stroke, although RIC is a safe procedure that can easily be implemented in hospital and pre-hospital settings, its clinical efficacy has yet to be proven (20). Furthermore, no biomarkers equivalent to CK-MB or high-sensitivity troponin T (41, 42) exist for ischemic stroke. Thus, only indirect evidence concerning RIC effects in brain ischemia is obtained from existing biomarkers and, as discussed, contradictory results have been reported in existing studies (68, 72).

**Table 1:** Summary of the key completed randomized controlled trials (published in English) on RIC with clinical outcomes in patients with acute STEMI or ischemic stroke.

	**References**	***n***	**Conditioning intervention**	**Clinical findings in the intervention vs. control group**
STEMI	Sloth et al. (50)	333	4 × 5-min 200 mmHg, arm (per-RIC)	Reduced rates of MACCE and all-cause mortality at a median of 3.8 years
	Gaspar et al. (49)	258	3 × 5-min 200 mmHg, leg (per-RIC)	Reduced in-hospital HF and lower risks of cardiac mortality and/or hospitalization for HF at a median of 2.1 years
	Stiermaier et al. (48)	696	3 × 5-min 200 mmHg, arm (per-RIC)	Reduced rates of MACE and HF at a median of 3.6 years
	Hausenloy et al. (51)	5,401	4 × 5-min 200 mmHg, arm (per-RIC)	No significant differences in cardiac mortality, hospitalization for HF, or MACCE at 12 months
Acute ischemic stroke	Hougaard et al. (66)	443	4 × 5-min 200 mmHg or 25 mmHg above SBP, arm (per-RIC)	No significant difference in mRS at 90 days
	An et al. (76)	68	5 × 5-min 180 mmHg, both arms (post-RIC)	Favorable recovery (mRS score 0–1) at 90 days in the post-RIC group (adjusted OR 9.85, 95% confidence interval 1.54–63.16; *p* = 0.016)
	Pico et al. (74)	188	4 × 5-min 110 mmHg above SBP, thigh (per-RIC)	No significant difference in mRS at 90 days
	He et al. (77)	49	4 × 5-min 200 mmHg, arm (post-RIC)	No significant difference in mRS nor NIHSS at 90 days

*HF, heart failure; MACCE, major adverse cardiac and cerebrovascular events; MACE, major adverse cardiac events; mRS, modified Rankin score; NIHSS, National Institute of Health Stroke Scale; OR, odds ratio; RIC, remote ischemic conditioning; SBP, systolic blood pressure; STEMI, ST-segment elevation myocardial infarction*.

Further research is needed to better characterize RIC patient responses. Besides those discussed above, putative biomarkers of RIC effects include autocoids (e.g., adenosine, endogenous opioids, or bradykinin), cytokines, and nitrites, but other humoral factors are yet to be better defined (24). The interest in characterizing such biomarkers is two-fold. Firstly, they may clarify the mechanisms underlying RIC. Secondly, they could confirm that a certain protocol is effective in triggering a RIC response, if a defined threshold of a hypothetical biomarker were reached. Defining the exact mechanisms mediating the effects of pre-, per-, or post-RIC will also be crucial to develop drugs or technological devices that may replace RIC. This is important for many reasons. Firstly, because the lack of conclusive evidence on RIC efficacy in terms of clinical outcomes in MI and ischemic stroke may be due to heterogenous protocols and different settings of application of RIC. A pharmacological treatment or an electronic device could allow the design of more standardized protocols, possibly allowing a benefit from RIC to emerge, even in human studies. Secondly, should this benefit be proven in humans, drugs or devices have the undisputable advantage that they can be administered or applied more quickly than the RIC protocol, which typically requires at least 40 min. One interesting perspective, since RIC cardiac protection appears to be at least partially mediated by the activation of vagal fibers (27, 40), is that vagal nerve stimulation may reproduce its effects in MI patients (26). Similarly, as vagus nerve activation has anti-inflammatory (78) and neuroprotective effects reducing cerebral infarct size (24, 79), vagal stimulation may mediate RIC effects in acute stroke (24). However, it is likely that isolated direct transcutaneous vagal stimulation does not trigger all protective processes that are instead activated by RIC protocols inducing muscle ischemia.

Measuring biomarkers mediating the RIC response may also help to stratify patients to identify those who could most benefit from remote ischemic post-conditioning in the days after an ischemic event.

A more stringent selection of included participants in future RIC clinical trials may boost effect size. For example, since RIC is believed to be most effective against cerebral reperfusion injury based on pre-clinical studies (24, 80), stroke clinical trials should focus on patients with a higher likelihood of successful reperfusion (24), who are thus exposed to the risk of reperfusion injury. Besides reperfusion, other risk factors include hypertension, cerebral vascular dysregulation, and late recanalization (81, 82).

Another interesting criterion of stratification of stroke patients may be the presence of collateral brain vessels, which can limit the extension of ischemic penumbra or border zone (83). Importantly, recent pre-clinical studies have found that RIC enhanced cerebral collateral circulation (84, 85). For this reason, the effects of RIC may be most evident in patients who can most benefit from the presence of vascular collaterals, such as those undergoing large vessel occlusion, who are also candidates for mechanical thrombectomy. For such reasons, testing RIC in this specific population may yield new, interesting results (20).

A possible cause of the discrepancy between results of pre-clinical and clinical studies on RIC is a difference in protocols. In fact, pre-clinical studies mostly employed RIC of the hindlimb, while upper limb RIC is usually performed in patients (24). It cannot be excluded that the higher proportion of muscle tissue undergoing RIC in the hindlimb may explain the greater efficacy of RIC in pre-clinical studies, especially if we consider that human patients might be elderly individuals with muscle deconditioning and comorbidities. Thus, leg RIC might be systematically used in future studies, as has previously been studied (41, 49, 74). Alternatively, a higher number of ischemia/reperfusion cycles and/or longer duration of the stimulus repetition might be more effective. Furthermore, post-conditioning long-term RIC protocols in patients are complicated by problems of compliance. Technological devices can also be used to document compliance and to ensure adherence to the protocol. Smart devices to monitor real-time post-conditioning, long-term RIC protocols, in association with smartphone applications, are currently being tested (86).

Finally, in both MI and acute ischemic stroke, it is crucial to apply RIC as soon as possible, even in the pre-hospital setting, to freeze the penumbra, or the border zone, thus possibly extending the time window for reperfusion therapies (20, 74). To make this possible, patients' early triage and stratification with pre-hospital scales is warranted (20). Importantly, it is possible to apply RIC in pre-hospital emergency settings, not only in an ambulance (21, 44), but also during air medical transportation (87). However, challenges to pre-hospital RIC administration include the need for dedicated personnel, if using a manual cuff, and the fact that average pre-hospital transport times may not be long enough to administer the full cycles. For instance, in one of the studies discussed above, transportation time was too short to administer four RIC cycles in 18% of patients (66). Once again, an automatic device can relieve both manpower and cognitive resources in the emergency setting, as it can be left in place once a pre-programmed protocol has been set. Interestingly, RIC can also be given in the emergency department, and even in the catheterization laboratory (88).

## Conclusions

RIC is “non-invasive, simple, safe, and cheap” (89), can be used alone or in combination with existing reperfusion therapies, and can be initiated in pre-hospital settings, but its clinical efficacy in MI and acute ischemic stroke patients remains to be proven (20, 47, 49–51, 66, 74). Further research is needed regarding the optimal time window and protocol for RIC application, its mechanisms and biomarkers, and the populations that could most benefit from this strategy in both cardiology and neurology settings.

## Author Contributions

LFS: design and writing. AA, ME, and FP: design and critical revision. All authors contributed to the article and approved the submitted version.

## Funding

This work was funded by the Center Hospitalier de Versailles.

## Conflict of Interest

The authors declare that the research was conducted in the absence of any commercial or financial relationships that could be construed as a potential conflict of interest.

## Publisher's Note

All claims expressed in this article are solely those of the authors and do not necessarily represent those of their affiliated organizations, or those of the publisher, the editors and the reviewers. Any product that may be evaluated in this article, or claim that may be made by its manufacturer, is not guaranteed or endorsed by the publisher.
